# Demarcation Laser Photocoagulation for Subclinical Retinal Detachment: Can Progression to Retinal Detachment Be Prevented?

**DOI:** 10.4274/tjo.galenos.2019.22844

**Published:** 2019-12-31

**Authors:** Nilüfer Koçak, Mahmut Kaya, Taylan Öztürk, Volkan Bolluk, Süleyman Kaynak

**Affiliations:** 1 Dokuz Eylül University Faculty of Medicine, Department of Ophthalmology, İzmir, Turkey; 2 Kadirli State Hospital, Clinic of Ophthalmology, Osmaniye, Turkey

**Keywords:** Clinical retinal detachment, first-line treatment, laser photocoagulation, subclinical retinal detachment

## Abstract

**Objectives:**

To describe results of demarcation laser photocoagulation in preventing progression of subclinical retinal detachment (SCRD).

**Materials and Methods:**

Twenty-one eyes of 20 patients with SCRD were included. All patients underwent a complete ophthalmological examination, spectral-domain optical coherence tomography, and color fundus photography. Ages at initial diagnosis ranged between 18 and 75 years (mean: 57.3±16.2 years). Patients followed for at least 6 months were included in the study. Periodic retinal examinations were performed over follow-up periods of 6-55 months using Goldmann three-mirror contact lens and sometimes semilunar mirror lens with scleral indentation.

**Results:**

Twelve patients (60%) were female, eight (40%) were male. The mean follow-up period was 24.3±15.2 months (6-55 months). Three (14.3%) eyes were pseudophakic. One patient was affected bilaterally, with both eyes each containing two separate areas of involvement. The SCRD was in the upper quadrant of 18 eyes (85.7%) and the lower quadrant in 3 eyes (14.3%), and was located in the temporal region 10 eyes (47.6%), the nasal quadrant in 4 eyes (19.1%), and in the upper quadrant (temporal-nasal) in 7 eyes (33.3%). Six eyes (28.6%) were found to have myopia greater than -3.0 diopters. Progression to clinical retinal detachment was observed in 4/21 SCRD eyes (19%). All eyes showing progression to clinical retinal detachment had >-3.0 diopter myopia and multiple retinal tears located in the upper quadrant.

**Conclusion:**

Demarcation laser photocoagulation should be kept in mind as a first-line treatment for eyes with SCRD. Laser photocoagulation is vital in preventing progression to rhegmatogenous retinal detachment in most patients. After this treatment, these patients should be followed closely.

## Introduction

There is no clear or sufficiently broad consensus among ophthalmologists regarding the term “subclinical retinal detachment” (SCRD). At present, SCRD is defined as rhegmatogenous retinal detachment that causes no change in visual acuity or visual field.^1^ Another commonly used definition is the presence of subretinal fluid that extends at least one disc diameter from the nearest break and no more than two disc diameters posterior to the equator.^2,3,4^ The real incidence and natural history of SCRD are unknown, as most patients are clinically asymptomatic. In the literature, clinical progression is reported in up to 50% of SCRDs.^5^ Therefore, these patients should be treated. If eyes with SCRD are left untreated, regular examination is imperative. SCRD may also lead to epiretinal membrane and/or cystoid macular edema.

Our main objective in this study was to evaluate the safety and effectiveness of demarcation laser photocoagulation in preventing the progression of SCRD to clinical retinal detachment. The secondary aim of the study was to investigate the presence of epiretinal membrane and/or cystoid macular edema in SCRD patients treated with demarcation laser photocoagulation.

## Materials and Methods

The study included 21 eyes of 20 patients who were followed for SCRD in the Retina Unit of the Dokuz Eylül University Faculty of Medicine, Department of Ophthalmology between October 2014 and May 2018 and had not previously received any treatment. The patients included in the study presented to the outpatient clinic due to refractive error and were diagnosed with SCRD upon fundus examination. The study was approved by Dokuz Eylül University Faculty of Medicine Ethics Committee (2018/29-35) and conducted in accordance with the accepted principles in the Declaration of Helsinki. All patients who volunteered to participate were informed in detail about the study and laser photocoagulation, and their written informed consent was obtained. Inclusion criteria were undergoing 360º laser photocoagulation for SCRD and being followed for at least 6 months after treatment. Exclusion criteria were reduced visual acuity or visual field loss associated with detachment upon initial examination, detachment that could not be encircled by 360° laser photocoagulation, history of other retinal surgery, and myopia greater than -8.0 D. The medical records of the patients included in the study were examined in detail and the data were recorded in full in SPSS.

All patients underwent a detailed eye examination prior to treatment. Best corrected visual acuity (BCVA) was measured by Snellen chart. Slit-lamp examination was performed and intraocular pressure was measured with Goldmann applanation tonometer. Fundus examination was performed on both affected and unaffected eyes with a Goldmann three-mirror contact lens. Color fundus photographs and spectral domain optical coherence tomography (SD-OCT) scans (Heidelberg HRA-OCT Spectralis, Heidelberg Engineering GmbH, Heidelberg, Germany) of the posterior segment were acquired for all patients prior to treatment.

**Figure 1:**
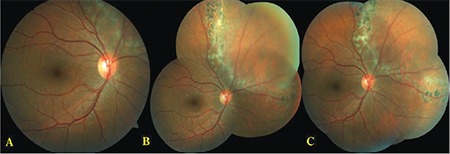
A) Color fundus photographs from a 19-year-old male patient (Patient 4) show subclinical retinal detachment secondary to a retinal tear in the peripheral upper nasal area of the right eye, B) appearance 1 month after demarcation laser photocoagulation, and C) appearance 6 months after demarcation laser photocoagulation. The entire SCRD and 360° laser demarcation could not be shown due to the difficulty of obtaining fundus photographs of the peripheral retina SRCD: Subclinical retinal detachment

**Figure 2:**
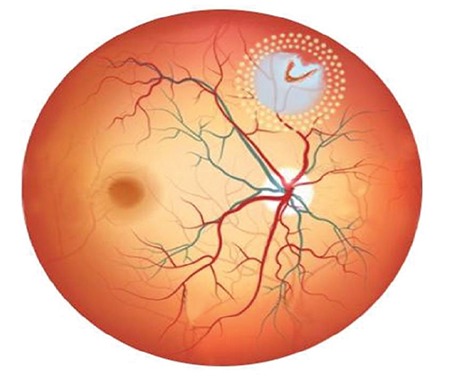
Schematic showing subclinical retinal detachment secondary to horseshoe retinal tear in the peripheral upper nasal area of the right eye and the 360° laser photocoagulation procedure

Argon laser photocoagulation was performed using the 360° demarcation technique surrounding the SCRD using a Goldmann three-mirror contact lens and, when necessary, a contact lens with scleral indentation (Figures 1 and 2). Additional laser photocoagulation was performed as necessary during follow-up. The argon green wavelength (514 µm) of the Zeiss Argon Laser system was used for photocoagulation. After instilling proparacaine hydrochloride 0.5% (Alcaine®) for topical anesthesia, the laser procedure was performed using a Goldmann three-mirror contact lens. Pulses were delivered with 200-micron spot diameter and 0.2-s duration, starting at energy of 180 mW and increasing the power when necessary.

Follow-up examinations were performed at 1, 3, and 6 weeks after laser photocoagulation, followed by monthly follow-ups for the remainder of the first 6 months. At all follow-up visits, patients underwent BCVA assessment with Snellen chart, anterior segment examination, intraocular pressure measurement, and detailed fundus examination using a Goldmann three-mirrored contact lens. Posterior segment color fundus photographs and SD-OCT scans were also acquired but not routinely at each visit.

### Statistical Analysis

The data obtained were recorded in SPSS 17.0 (SPSS Inc, Chicago, IL, USA). Means and standard deviations were calculated for all the data.

## Results

The patients’ demographic data, ophthalmic findings, and follow-up times are summarized in Table 1. Of the 20 patients with SCRD, 12 (60%) were female and 8 (40%) were male. The mean age was 57.3±16.2 years (18-75 years) and mean follow-up time was 24.3±15.2 months (6-55 months). Three eyes (14.3%) were pseudophakic. Axial length of the eyes was 22-27 mm. Of the 21 affected eyes, 11 (52%) were right eyes and 10 (48%) were left eyes. One patient (patient: 9, Table 1) had bilateral involvement, with two separate areas affected in each eye. In terms of retinal quadrants affected, SCRD was in the upper quadrant in 18 eyes (85.7%) and the lower quadrant in 3 eyes (14.3%), and was located in the temporal quadrant in 10 eyes (47.6%), the nasal quadrant in 4 eyes (19.1%), and both the temporal and nasal quadrants in 7 eyes (33.3%). Etiology of SCRD was secondary to lattice degeneration in 16 eyes, secondary to posterior vitreous detachment (horseshoe tear) in 4 eyes, and secondary to an atrophic hole in 1 eye.

Myopia greater than -3.0 D was detected in 6 (28.6%) of the eyes with SCRD. Epiretinal membrane and/or cystoid macular edema occurred in 8 (38%) of the eyes included in the study (Table 1). In the 3 eyes (14.3%) that developed cystoid macular edema, complete regression was observed with topical nepafenac 3 times a day used for a mean of 3 months. Progression to clinical retinal detachment was observed in 4 (19%) of the 21 eyes during follow-up, and these patients underwent pars plana vitrectomy (PPV). All of the eyes with progression to clinical retinal detachment had greater than -3.0 D myopia and multiple retinal breaks in the upper quadrant. No recurrence was observed in the eyes that underwent PPV. No major complications related to demarcation laser photocoagulation were observed.

## Discussion

The term SCRD was first used in 1952 by Schepens^6^ to describe cases in which a diagnosis of clinical retinal detachment could not be established using the usual investigation methods. The methods available at that time included direct ophthalmoscopy and sometimes slit-lamp biomicroscopy and visual field examination. In 1958, Schepens^1^ defined SCRD as retinal detachment that does not cause changes in the patient’s visual field or visual acuity. The criterion used in this second definition formed the basis of the term SCRD as currently used. In 1973, Davis^7^ described SCRD in anatomical terms, delimiting and thus substantially refining its definition. At present, there is no complete consensus regarding the term SCRD.

Detachments that do not cause visual field defects or reduce visual acuity may not be noticed by patients. In such cases, the retinal detachment is either self-limited to the demarcation line or progresses to clinical retinal detachment. Because patients with SCRD are asymptomatic and do not receive medical treatment, the real incidence or natural history of SCRD cannot be determined. There are few studies in the literature reporting the natural course of SCRD. In a study by Byer^8^ including 17 eyes with asymptomatic retinal tears and long-term follow-up, 18 SCRD areas were monitored without treatment. In the natural course of SCRD in these eyes, 11% progressed from SCRD to clinical retinal detachment. In the same study, 59% of the SCRD areas were in the lower retinal quadrants and 90.9% of the eyes had involvement in the temporal half of the retina. This localization may have contributed to SCRD being preserved as subclinical and remaining asymptomatic in terms of visual field defects. Brod et al.^9^ followed 31 eyes of 28 patients with asymptomatic rhegmatogenous retinal detachment for a mean of 3.4 years and observed progression of detachment in only 6% of the eyes.

The literature is not clear regarding how to approach SCRD or the necessity of treatment.^8,9,10,11,12^ Although these detachments exhibit different clinical states during their natural course, they may progress to a symptomatic condition. In symptomatic detachments that develop from SCRD, the former detachment line is seen in most patients.^9^ Because there is insufficient data on the natural course of SCRD, the risk of their progression to symptomatic retinal detachment is not fully known. When deciding whether or not to treat a patient, treatment should be considered in patients who need to be physically active; those who have superiorly located, horseshoe, or multiple retinal tears; and most importantly, those who develop symptomatic retinal detachment.^5^ Intraocular surgery to treat SCRD carries the risk of postoperative decrease in vision. As many patients with SCRD have ≥0.8 (Snellen) visual acuity, substantial reductions in visual acuity may be observed following surgery.

Since the development of microincision approaches (23-, 25-, and 27-gauge), wide-angle imaging systems, high-speed cutters, and better illumination methods, PPV is now preferred for the treatment of retinal detachment.^13^Scleral buckling surgery (cerclage band), a minimal surgical method, can also yield successful outcomes in suitable patients. However, this procedure is less preferred because some surgeons working in this field have poor command of the indirect ophthalmoscope, and there is a lack of time and training programs for cerclage training.^14^ Different therapeutic approaches to SCRD and clinical retinal detachment can be observed among physicians. The decision to pursue invasive treatment or to monitor SCRD is not urgent, and surgical interventions may not be necessary in primary treatment as long as there is no clinical retinal detachment in the patient’s fellow eye. We preferred demarcation laser photocoagulation as a more conservative therapeutic approach in our SCRD patients and achieved successful outcomes with this noninvasive treatment.

There is evidence that adhesion between the neural retina and retinal pigment epithelium begins to develop 24 hours after the treatment of laser photocoagulation.^15^ However, it takes 3 to 14 days to reach maximum strength.^16^ If laser photocoagulation can be successfully applied in 360° surrounding the detachment, it is a non-invasive, simple, and effective treatment option. In all of our patients, the detachments were encircled with 3-4 rows of 360° laser photocoagulation and monitored closely. Epiretinal membrane and/or cystoid macular edema was detected in 38% of the eyes, but none of the eyes required surgical treatment for epiretinal membrane. The 3 eyes (14.3%) that developed cystoid macular edema, were treated with topical nepafenac 3 times a day, and regression was observed within an average of 3 months. SCRD progression to clinical retinal detachment occurred in 19% (4 eyes) during the long-term follow-up of our patients. We noted that the eyes in our study that progressed to clinical retinal detachment and underwent PPV had multiple (≥2) retinal tears and SCRD located in the upper quadrant.

In patients with SCRD, demarcation laser photocoagulation should be kept in mind as a primary treatment to avoid the possible complications of intraocular surgery. Demarcation laser photocoagulation is of great importance in preventing progression to clinical retinal detachment in most patients. Patients should be monitored closely after demarcation laser photocoagulation therapy. The patient group most at risk for progression to clinical retinal detachment includes those with multiple retinal tears, upper quadrant involvement, and extensive subretinal fluid, and these patients should therefore be monitored more closely and carefully.

## Figures and Tables

**Table 1:**
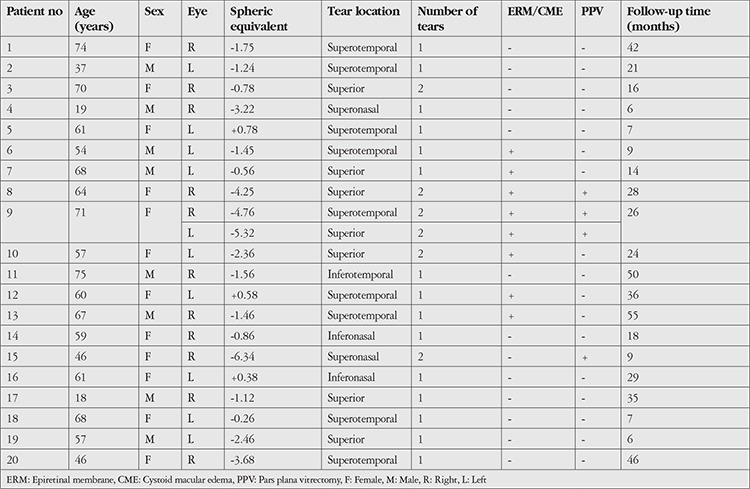
Demographic data, ocular findings, and follow-up periods of the patients
